# Obesity- and lipid-related indices as a predictor of obesity metabolic syndrome in a national cohort study

**DOI:** 10.3389/fpubh.2023.1073824

**Published:** 2023-02-14

**Authors:** Jiaofeng Gui, Yuqing Li, Haiyang Liu, Lei-lei Guo, Jinlong Li, Yunxiao Lei, Xiaoping Li, Lu Sun, Liu Yang, Ting Yuan, Congzhi Wang, Dongmei Zhang, Huanhuan Wei, Jing Li, Mingming Liu, Ying Hua, Lin Zhang

**Affiliations:** ^1^Department of Graduate School, Wannan Medical College, Wuhu, Anhui, China; ^2^Student Health Center, Wannan Medical College, Wuhu, Anhui, China; ^3^Department of Surgical Nursing, School of Nursing, Jinzhou Medical University, Jinzhou, Liaoning, China; ^4^Department of Occupational and Environmental Health, Key Laboratory of Occupational Health and Safety for Coal Industry in Hebei Province, School of Public Health, North China University of Science and Technology, Tangshan, Hebei, China; ^5^Obstetrics and Gynecology Nursing, School of Nursing, Wannan Medical College, Wuhu, Anhui, China; ^6^Department of Emergency and Critical Care Nursing, School of Nursing, Wannan Medical College, Wuhu, Anhui, China; ^7^Department of Internal Medicine Nursing, School of Nursing, Wannan Medical College, Wuhu, Anhui, China; ^8^Department of Pediatric Nursing, School of Nursing, Wannan Medical College, Wuhu, Anhui, China; ^9^Department of Surgical Nursing, School of Nursing, Wannan Medical College, Wuhu, Anhui, China; ^10^Rehabilitation Nursing, School of Nursing, Wanna Medical College, Wuhu, Anhui, China

**Keywords:** metabolic syndrome, lipids, obesity, national cohort study, middle-aged and elderly Chinese

## Abstract

**Objective:**

Metabolic syndrome is a common condition among middle-aged and elderly people. Recent studies have reported the association between obesity- and lipid-related indices and metabolic syndrome, but whether those conditions could predict metabolic syndrome is still inconsistent in a few longitudinal studies. In our study, we aimed to predict metabolic syndrome by obesity- and lipid-related indices in middle-aged and elderly Chinese adults.

**Method:**

A national cohort study that consisted of 3,640 adults (≥45 years) was conducted. A total of 13 obesity- and lipid-related indices, including body mass index (BMI), waist circumference (WC), waist-to-height ratio (WHtR), conicity index (CI), visceral adiposity index (VAI), Chinese visceral adiposity index (CVAI), lipid accumulation product (LAP), a body shape index (ABSI), body roundness index (BRI), and triglyceride glucose index (TyG-index) and its correlation index (TyG-BMI, TyG-WC, and TyG-WHtR), were recorded. Metabolic syndrome (MetS) was defined based on the criteria of the National Cholesterol Education Program Adult Treatment Panel III (2005). Participants were categorized into two groups according to the different sex. Binary logistic regression analyses were used to evaluate the associations between the 13 obesity- and lipid-related indices and MetS. Receiver operating characteristic (ROC) curve studies were used to identify the best predictor of MetS.

**Results:**

A total of 13 obesity- and lipid-related indices were independently associated with MetS risk, even after adjustment for age, sex, educational status, marital status, current residence, history of drinking, history of smoking, taking activities, having regular exercises, and chronic diseases. The ROC analysis revealed that the 12 obesity- and lipid-related indices included in the study were able to discriminate MetS [area under the ROC curves (AUC > 0.6, *P* < 0.05)] and ABSI was not able to discriminate MetS [area under the ROC curves (AUC < 0.6, *P* > 0.05)]. The AUC of TyG-BMI was the highest in men, and that of CVAI was the highest in women. The cutoff values for men and women were 187.919 and 86.785, respectively. The AUCs of TyG-BMI, CVAI, TyG-WC, LAP, TyG-WHtR, BMI, WC, WHtR, BRI, VAI, TyG index, CI, and ABSI were 0.755, 0.752, 0.749, 0.745, 0.735, 0.732, 0.730, 0.710, 0.710, 0.674, 0.646, 0.622, and 0.537 for men, respectively. The AUCs of CVAI, LAP, TyG-WC, TyG-WHtR, TyG-BMI, WC, WHtR, BRI, BMI, VAI, TyG-index, CI, and ABSI were 0.687, 0.674, 0.674, 0.663, 0.656, 0.654, 0.645, 0.645, 0.638, 0.632, 0.607, 0.596, and 0.543 for women, respectively. The AUC value for WHtR was equal to that for BRI in predicting MetS. The AUC value for LAP was equal to that for TyG-WC in predicting MetS for women.

**Conclusion:**

Among middle-aged and older adults, all obesity- and lipid-related indices, except ABSI, were able to predict MetS. In addition, in men, TyG-BMI is the best indicator to indicate MetS, and in women, CVAI is considered the best hand to indicate MetS. At the same time, TyG-BMI, TyG-WC, and TyG-WHtR performed better than BMI, WC, and WHtR in predicting MetS in both men and women. Therefore, the lipid-related index outperforms the obesity-related index in predicting MetS. In addition to CVAI, LAP showed a good predictive correlation, even more closely than lipid-related factors in predicting MetS in women. It is worth noting that ABSI performed poorly, was not statistically significant in either men or women, and was not predictive of MetS.

## Introduction

**Figure 1:**
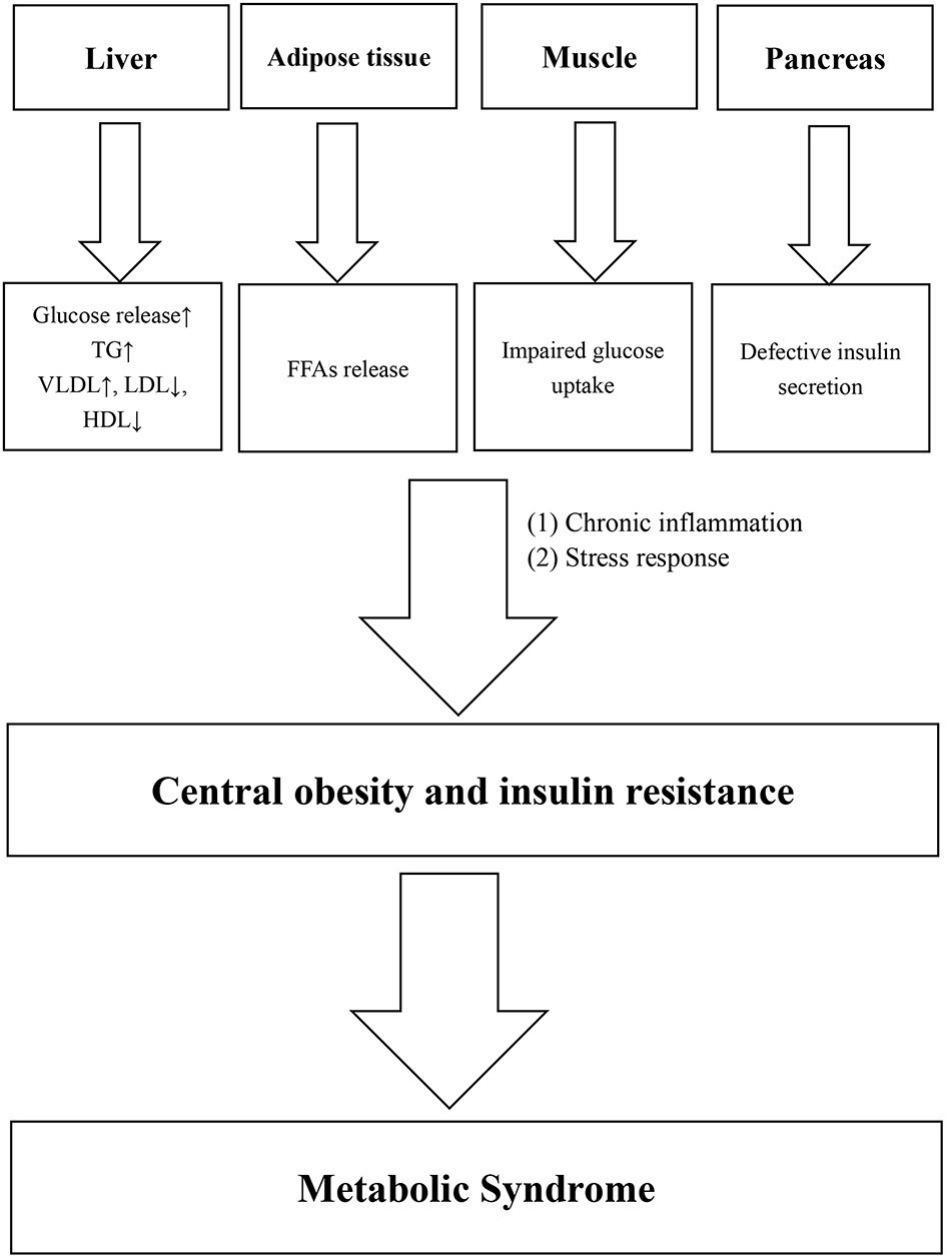
Pathophysiological changes of metabolic syndrome.

Metabolic syndrome (MetS) is a common condition among middle-aged and elderly people. The prevalence was 19.2% in men and 27.0% in women in China (1). The MetS consists of five major components, namely, central obesity, elevated triglyceride (TG) levels, low high-density lipoprotein cholesterol (HDL-C) levels, elevated BP, and elevated fasting plasma glucose (FPG) levels (2). Central obesity (3) and insulin resistance are considered the main pathogenesis of MetS, while chronic inflammation (4, 5) and stress response (6) also play important roles. Various pathological damages, such as glucose and lipid dysfunction in the liver, free fatty acid (FFAs) release from adipose tissues, impaired glucose uptake in muscle, and defective insulin secretion in the pancreas, can lead to central obesity or insulin resistance. At the same time, chronic inflammation and stress reaction also play an important role in this process. Figure 1 shows the main physiological changes of MetS.

Several studies (7, 8) have shown that MetS causes different degrees of damage to the cardiovascular system, digestive system, and endocrine system. Obesity can result in a significantly increased risk of death from other conditions such as cardiovascular disease, digestive tract diseases, endocrine diseases, or cancer (9). A cross-sectional analysis of 59 studies in the Middle East in 2017 found that the comprehensive estimated prevalence of MetS was 25%, and it was also a significant cause of stroke, coronary heart disease, and cardiovascular diseases (10). Gluvic et al. (11) found that for patients with cardiovascular disease, the mortality rate of patients with MetS is much higher than that of patients without MetS. Meanwhile, MetS may lead to cancer (12).

A meta-analysis of global data from 28 million subjects showed that the worldwide prevalence of MetS ranged from 12.5 to 31.4%, with the Eastern Mediterranean region and the Americas showing significantly higher prevalence than other regions (13). Among Europeans between the ages of 30 and 89 years, the incidence of MetS in men is 41%, while that in women is 38% lower than that in men (14). Adult white men were more likely to have abdominal obesity than other races, whereas white women lacked the opposite, which may explain this phenomenon (15). In the Asia Pacific region, the Philippines reported a minimum prevalence of 11.9%, while Pakistan reported a MetS prevalence of 49.0% (16). Possible factors that affect the large difference in the incidence of MetS in the Asia-Pacific region include smoking, alcohol consumption, lack of physical activity, and unhealthy diet (17). We also observed an increasing trend in the prevalence of MetS in China (13.7% in 2000–2001 and 21.3% in 2009) (18, 19). A cross-sectional study of 8,040 community residents in Jiangsu Province, China, showed that the prevalence of MetS was 35.2% in 2014 (20).

Middle-aged and older adults often suffer from a variety of chronic diseases. At the same time, their body immunity was low. Therefore, MetS was more likely to cause more serious consequences or even death. Consequently, it was necessary to find a practical index for predicting MetS. Mounting evidence demonstrates that obesity- and lipid-related indices could be associated with MetS. The current meta-analysis attempts to estimate the strength of the relationship between obesity- and lipid-related indices and MetS. A recent meta-analysis conducted with 155 patients by Ecder and Sasak (21) found that waist-to-height ratio (WHtR) in women [area under the ROC curve (AUC) = 0.815, 95% CI = 0.687–0.942] and waist circumference (WC) in men (AUC = 0.826, 95% CI = 0.741–0.911) prove to be better than other anthropometric measures for predicting MetS in renal transplant recipients. Another meta-analysis performed for the 232 participants by Khan et al. (22) found that WHtR and AVI performed best in predicting MetS. In addition, a number of meta-analysis studies (23–25) also proved the association between obesity- and lipid-related indices and MetS. Although the meta-analysis used cohort and cross-sectional studies to confirm the potential association between obesity- and lipid-related indicators and MetS, the strength of the prediction of obesity- and lipid-related indicators for MetS was not analyzed in the same field. In addition, most meta-analyses included only Western participants, and some studies considered other ethnic groups, such as Asians. Therefore, further studies in middle-aged and older adults in China are needed to determine the strength of the association between obesity- and lipid-related indices in predicting MetS in Chinese participants.

To fill these gaps, we used longitudinal data from a nationally representative sample of participants of community residents in China who were ≥45 years old and explored to examine the relationship between obesity- and lipid-related indices and incidence of MetS. In addition, this study explored the stability of the association between components of obesity- and lipid-related indicators and MetS by controlling for potential confounders.

## Materials and methods

### Participants

**Figure 2:**
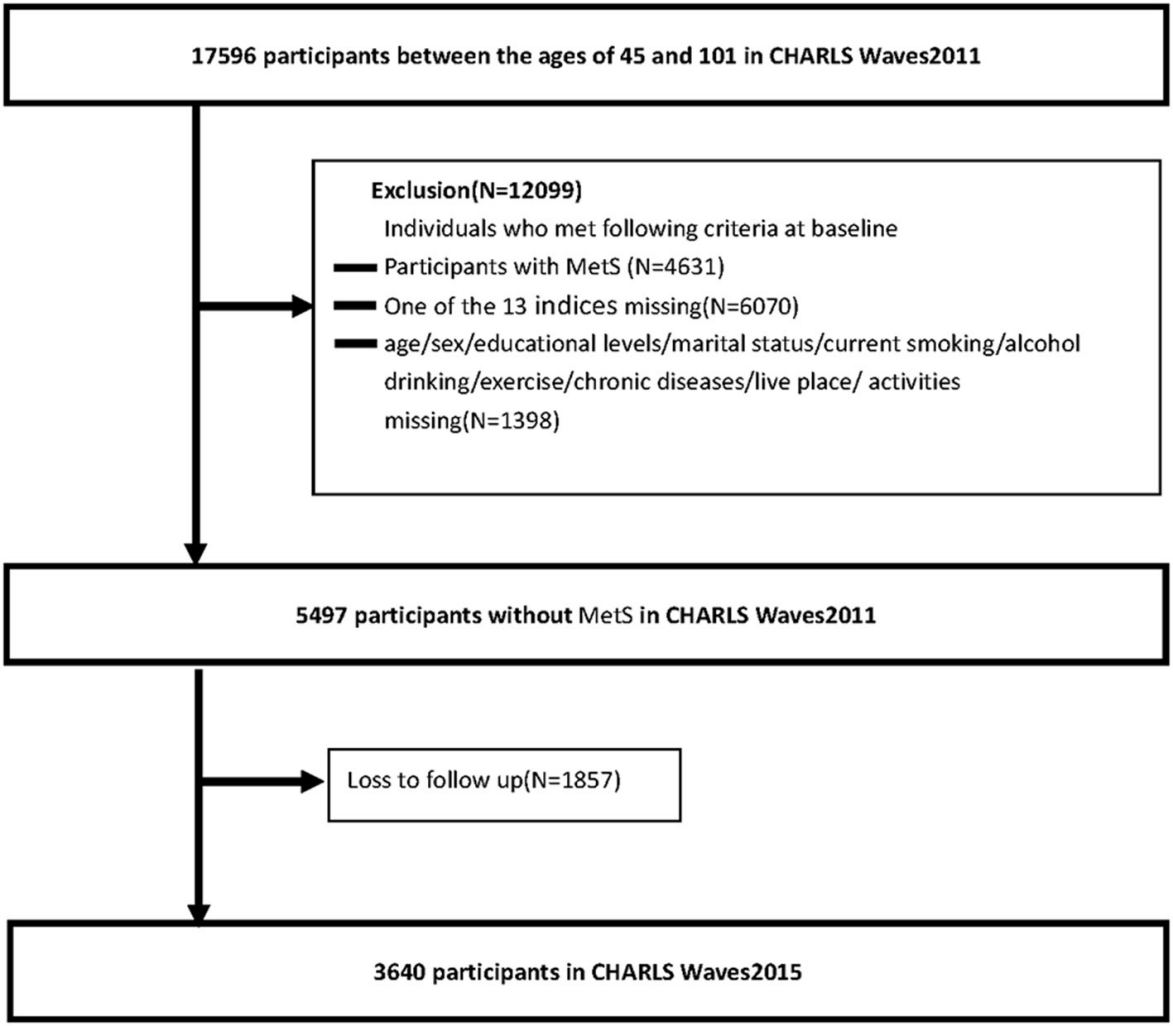
Flowchart of the study participants.

Participants in this national cohort study were Chinese community residents aged older than 45 years who participated in the China Health and Retirement Longitudinal Study (CHARLS) survey. CHARLS is a nationally representative longitudinal survey of middle-aged and elderly Chinese adults and their spouses. The CHARLS began in 2011 with a cohort of 17,596 participants between 45 and 101 (Waves1) years and collected data in 2013 (Waves2) and 2015 (Waves3). Participants will undergo face-to-face, computer-assisted personal interviews (CAPIs), and structured questionnaires every 2 years. The study used data from participants in Waves1 and Waves3. We excluded individuals who met any of the following criteria at baseline: (1) participants with MetS, (2) one of the 13 indices missing, and (3) age/sex/educational level/marital status/current smoking/alcohol drinking/exercise/chronic diseases/live place/activities missing. In addition, we excluded participants with no follow-up data. The numbers of individuals who completed both the baseline and follow-up surveys were 3,640 for the long term (2011–2015). Figure 2 shows a flow diagram of the study individuals, follow-up, and missed follow-up.

### Metabolic syndrome symptom

The NCEP ATP III (2005) (26, 27) proposed the definition and diagnostic criteria of MetS. According to the standard Chinese definition (28, 29), components of MetS are divided into five categories:

1. The waist circumference (WC) of central obesity is defined as ≥80 cm for women and ≥90 cm for men.
2. Elevated TG levels: TG levels of ≥150 mg/dl.
3. Low HDL-C levels: HDL-C levels of <40 mg/dl for men and <50 mg/dl for women.
4. Elevated BP: systolic blood pressure (SBP) of ≥130 mmHg and/or diastolic blood pressure (DBP) of ≥85 mmHg or using antihypertensive therapy.
5. Elevated FPG levels: FPG levels of ≥100 mg/dl or using antidiabetic medications or self-reported medical history of diabetes.

When three of the five listed characteristics are present, a diagnosis of MetS can be made.

### Covariates

In this study, we classified the participants into two groups according to gender. According to the International Diabetes Federation (IDF) (30), age, obesity, and a lack of exercise are all risk factors for MetS. Yang et al.'s (31) research shows that there is a strong link between MetS and age and place of residence. At the same time, smoking and drinking alcohol have also been confirmed to lead to MetS (32, 33). According to the previous standards (34, 35), we classified 14 chronic diseases, which were divided into three categories according to the number of chronic diseases: 0, 1–2, and 3–14. Referring to our previous research (34, 36–42), we formulated the basis for choosing the 13 obesity- and lipid-related indices. These bases included age, education, marriage, residence, drinking, smoking, activities, exercise, and chronic diseases. The classification of nine covariates is shown as follows:

1. Age: (1) below 45–54 years, (2) 55–64 years, (3) 65–74 years, and (4) above 75 years.
2. Education level: (1) illiterate, (2) less than elementary school, (3) high school, and (4) above vocational school.
3. Marital status: (1) single (divorced, never married, widowed, or separated) and (2) married.
4. Current residence: (1) rural and (2) urban.
5. Current smoking: (1) current smokers, (2) former smokers, and (3) never smokers.
6. Alcohol drinking: (1) never drinker, (2) less than once a month, and (3) more than once a month.
7. Taking activities: (1) yes and (2) no.
8. Having regular exercises: (1) no physical exercise, (2) less than regular physical exercise, and (3) regular physical exercise.
9. Chronic diseases: (1) 0, (2) 1–3, and (3) 4–16.

### Measurements

At the end of exhalation and before inhalation, the circumference of the midpoint line is measured between the lowest point of the rib and the upper edge of the iliac crest with a soft ruler, which is called WC (43). It is worth noting that the remaining 12 indicators need to be calculated. BMI is calculated as the body mass (kg) divided by the square of the body height (m) (44). WHtR is defined as the WC (m) divided by the height (m) (45). The visceral adiposity index (VAI), Chinese visceral adiposity index (CVAI), lipid accumulation product (LAP), and triglyceride glucose index (TyG-index) are calculated to obtain TG and HDL through invasive examination. VAI is calculated by four indices, namely, WC, BMI, TG, and HDL (46). It is worth noting that the figures in the calculation formulas of VAI for men and women are slightly different, but they are all completed by these four same measurement indices. A body shape index (ABSI) is determined by a comprehensive calculation of WC, BMI, and height (47). Body roundness index (BRI) is calculated using two basic metrics, i.e., WC and height (48). LAP is calculated slightly differently by subtracting a number (males: 65 cm, females: 58 cm) from WC and multiplying by TG (49). The measurement method of CI is completed by WC, weight, and height (50). CVAI is calculated based on VAI to develop a more suitable measurement of the Chinese people (51). The calculation result with TG and glucose is defined as the TyG index (52). At the same time, TyG-BMI, TyG-WC, and TyG-WHtR are obtained by multiplying TyG with BMI, WC, and WHtR (53–55). The 12 indices except WC are calculated as follows:

1. BMI = Weight/Height^2^
2. WHtR = WC/Height
3. Males: VAI $=\frac{WC}{39.68+\left(1.88\times BMI\right)}\times \frac{TG}{1.03}\times \frac{1.31}{HDL}$ Females: VAI $=\frac{WC}{36.58+\left(1.89\times BMI\right)}\times \frac{TG}{0.81}\times \frac{1.52}{HDL}$
4. ABSI $=\frac{WC}{Height^{\frac{1}{2}}\times BMS^{\frac{2}{3}}}$
5. BRI $=364.2-365.5\sqrt{1-\left(\frac{WC÷{\left(2\pi \right)}^{2}}{{\left(0.5\times Height\right)}^{2}}\right)}$
6. Males: LAP = [WC (cm) – 65] × TG (mmol/L) Females: LAP = [WC (cm) – 58] × TG (mmol/L)
7. CI $=\frac{WC\left(m\right)}{0.019\sqrt{\frac{\text{weight}\left(\text{kg}\right)}{\text{height}\left(\text{m}\right)}}}$
8. Males: CVAI = −267.93 + 0.68 × age + 0.03 × BMI (kg/m^2^) + 4.00 × WC (cm) + 22.00 × Log_10_TG (mmol/L) – 16.32 × HDL-C (mmol/L) Females: CVAI = −187.32 + 1.71 × age + 4.32 × BMI (kg/m^2^) + 1.12 × WC (cm) + 39.76 × Log_10_TG (mmol/L) – 11.66 × HDL-C (mmol/L)
9. TyG index = Ln [(TG (mg/dl) × glucose (mg/dl)/2)]
10. TyG-BMI = TyG × BMI
11. TyG-WC = TyG × WC
12. TyG-WHtR = TyG × WHtR.

### Statistical analysis

All data were analyzed using the SPSS software, version 25.0 (IBM SPSS, Armonk, NY, USA). The chi-square test was used to classify variables, and the *t*-test was used to determine the significant differences between continuous variables. Using these two methods, we tested the degree of correlation between covariates and 13 obesity- and lipid-related indices and gender in Table 1. In Table 2, the degree of association between these indices and the presence or absence of MetS was examined. The receiver operating characteristic (ROC) curve was drawn and AUC was calculated to check the ability of these indices to identify the MetS. The cutoff points were selected using the Youden index (sensitivity + specificity – 1). The likelihood ratio was determined, and the effect size (ES) and odds ratio (OR) for each metric were calculated to determine the predictor for each metric. The *p*-value obtained by the statistical significance test was generally considered statistically significant when *P* < 0.05 and extremely significant when *P* < 0.001. According to optimal cutoff values of the 13 obesity- and lipid-related indices, they were divided into two categorical variables, and OR and 95% CI of each obesity- and lipid-related indices with MetS components were recalculated. After adjusting for age, education level, marital status, current residence, current smoking, alcohol drinking, taking activities, doing regular exercise, and chronic diseases, OR and 95% CI of each obesity- and lipid-related indices with MetS components were calculated.

**Table 1:** Characteristics of participants with full samples in baseline (*N* = 3,640).

**Variables**	**Male**	**Female**	**Total**	***t*/*X*^2^**	***P***
***N***	***N* = 1,968**	***N* = 1,672**	***N* = 3,640**		
**Age (years)**				130.285	0.000
45–54	547 (27.79)	734 (43.90)	1,281 (35.19)	
55–64	810 (41.16)	637 (38.10)	1,447 (39.75)	
65–74	486 (24.70)	236 (14.11)	722 (19.84)	
≥75	125 (6.35)	65 (3.89)	190 (5.22)	
**Education**				366.202	0.000
Illiterate	274 (13.92)	700 (41.87)	974 (26.76)	
Less elementary	1,476 (75.00)	872 (52.15)	2,348 (64.51)	
High school	148 (7.52)	77 (4.61)	225 (6.18)	
Above vocational	70 (3.56)	23 (1.38)	93 (2.55)	
**Marital status**				9.547	0.002
Married	1,794 (91.16)	1,472 (88.04)	3,266 (89.73)	
Single	174 (8.84)	200 (11.96)	374 (10.27)	
**Current residence**				0.916	0.339
Rural	1,868 (94.92)	1,575 (94.20)	3,443 (94.59)	
Urban	100 (5.08)	97 (5.80)	197 (5.41)	
**Current smoking**				1,805.457	0.000
No	459 (23.32)	1,564 (93.54)	2,023 (55.58)	
Former smoke	285 (14.48)	17 (1.02)	302 (8.30)	
Current smoke	1,224 (62.20)	91 (5.44)	1,315 (36.13)	
**Alcohol drinking**				821.328	0.000
No	820 (41.67)	1,459 (87.26)	2,279 (62.61)	
Less than once a month	221 (11.23)	80 (4.78)	301 (8.27)	
More than once a month	927 (47.10)	133 (7.95)	1,060 (29.12)	
**Taking activities**				3.174	0.075
No	994 (50.51)	894 (53.47)	1,888 (51.87)	
Yes	974 (49.49)	778 (46.53)	1,752 (48.13)	
**Having regular exercises**				1.011	0.603
No exercise	1,213 (61.64)	1,004 (60.05)	2,217 (60.91)	
Less than exercises	400 (20.33)	358 (21.41)	758 (20.82)	
Regular exercises	355 (18.04)	310 (18.54)	665 (18.27)	
**Chronic diseases (counts)**				0.544	0.762
0	704 (35.77)	584 (34.93)	1,288 (35.38)	
1–2	974 (49.49)	848 (50.72)	1,822 (50.05)	
3–14	290 (14.74)	240 (14.35)	530 (14.56)	
WC	81.47 ± 7.67	80.91 ± 9.00	81.22 ± 8.32	1.988	0.047
BMI	21.91 ± 3.05	22.64 ± 3.65	22.25 ± 3.36	−6.471	0.000
WHtR	0.50 ± 0.05	0.53 ± 0.06	0.51 ± 0.05	−17.260	0.000
VAI	2.43 ± 1.58	3.27 ± 1.76	2.82 ± 1.71	−15.184	0.000
ABSI	8.18 ± 0.54	8.22 ± 0.59	8.20 ± 0.56	−2.238	0.025
BRI	3.39 ± 0.88	4.00 ± 1.20	3.67 ± 1.08	−17.310	0.000
LAP	17.93 ± 13.12	24.27 ± 13.42	20.84 ± 13.62	−14.351	0.000
CI	1.25 ± 0.08	1.27 ± 0.09	1.26 ± 0.08	−4.567	0.000
CVAI	75.67 ± 34.77	79.31 ± 31.81	77.34 ± 33.49	−3.291	0.001
TyG index	8.37 ± 0.47	8.36 ± 0.42	8.37 ± 0.45	0.936	0.349
TyG-BMI	183.58 ± 28.10	189.3 ± 32.26	186.21 ± 30.21	−5.661	0.000
TyG-WC	682.62 ± 78.37	676.48 ± 82.91	679.8 ± 80.53	2.294	0.022
TyG-WHtR	4.19 ± 0.47	4.43 ± 0.54	4.30 ± 0.52	−14.516	0.000

The data are presented either as the mean ± SD or *n* (%).

WC, waist circumference; BMI, body mass index; WHtR, waist-to-height ratio; VAI, visceral adiposity index; ABSI, a body shape index; BRI, body roundness index; LAP, lipid accumulation product; CVAI, cardio-ankle vascular index; CI, conicity index; TyG, triglyceride and glucose index; TyG-BMI, TyG related to BMI; TyG-WC, TyG related to WC; TyG-WHtR, TyG related to WHtR.

**Table 2:** Follow-up characteristics of the study participants with and without MetS by sex in 2015.

**Variables**	**Male (*****N*** = **1,968)**		***t*/*X*^2^**	***P***	**Female (*****N*** = **1,672)**		***t*/*X*^2^**	***P***
	**With MetS**	**Without MetS**			**With MetS**	**Without MetS**		
	***N*** = **253**	***N*** = **1,715**			***N*** = **418**	***N*** = **1,254**		
**Age (years)**			3.512	0.319			14.133	0.003
45–54	79 (31.23)	468 (27.29)			167 (39.95)	567 (45.22)		
55–64	104 (41.11)	706 (41.17)			158 (37.80)	479 (38.20)		
65–74	52 (20.55)	434 (25.31)			65 (15.55)	171 (13.64)		
≥75	18 (7.11)	107 (6.24)			28 (6.70)	37 (2.95)		
**Education**			7.024	0.071			1.107	0.775
Illiterate	32 (12.65)	242 (14.11)			168 (40.19)	532 (42.42)		
Less elementary	184 (72.73)	1,292 (75.34)			227 (54.31)	645 (51.44)		
High school	21 (8.30)	127 (7.41)			18 (4.31)	59 (4.70)		
Above vocational	16 (6.32)	54 (3.15)			5 (1.20)	18 (1.44)		
**Marital status**			0.008	0.930			4.362	0.037
Married	231 (91.30)	1,563 (91.14)			356 (85.17)	1,116 (89.00)		
Single	22 (8.70)	152 (8.86)			62 (14.83)	138 (11.00)		
**Current residence**			2.489	0.115			1.317	0.251
Rural	235 (92.89)	1,633 (95.22)			389 (93.06)	1,186 (94.58)
Urban	18 (7.11)	82 (4.78)			29 (6.94)	68 (5.42)
**Current smoking**			4.020	0.134			0.184	0.912
No	58 (22.92)	401 (23.38)			390 (93.30)	1,174 (93.62)		
Former smoke	47 (18.58)	238 (13.88)			5 (1.20)	12 (0.96)		
Current smoke	148 (58.5)	1,076 (62.74)			23 (5.50)	68 (5.42)		
**Alcohol drinking**			4.050	0.132			1.770	0.413
No	103 (40.71)	717 (41.81)			370 (88.52)	1,089 (86.84)		
Less than once a month	20 (7.91)	201 (11.72)			15 (3.59)	65 (5.18)		
More than once a month	130 (51.38)	797 (46.47)			33 (7.89)	100 (7.97)		
**Taking activities**			0.416	0.519			1.696	0.193
No	123 (48.62)	871 (50.79)			212 (50.72)	682 (54.39)		
Yes	130 (51.38)	844 (49.21)			206 (49.28)	572 (45.61)		
**Having regular exercises**			0.323	0.851			0.393	0.821
No exercise	156 (61.66)	1,057 (61.63)			251 (60.05)	753 (60.05)		
Less than exercises	54 (21.34)	346 (20.17)			86 (20.57)	272 (21.69)		
Regular exercises	43 (17.00)	312 (18.19)			81 (19.38)	229 (18.26)		
**Chronic diseases (counts)**			2.387	0.303			3.767	0.152
0	100 (39.53)	604 (35.22)			130 (31.10)	454 (36.20)		
1–2	114 (45.06)	860 (50.15)			222 (53.11)	626 (49.92)		
3–14	39 (15.42)	251 (14.64)			66 (15.79)	174 (13.88)		
WC	87.01 ± 8.09	80.65 ± 7.26	−12.798	0.000	84.52 ± 9.02	79.71 ± 8.68	−9.703	0.000
BMI	23.84 ± 3.02	21.63 ± 2.95	−11.099	0.000	23.87 ± 3.73	22.23 ± 3.53	−8.081	0.000
WHtR	0.53 ± 0.05	0.50 ± 0.04	−11.478	0.000	0.55 ± 0.06	0.52 ± 0.06	−9.275	0.000
VAI	3.15 ± 1.83	2.32 ± 1.51	−6.884	0.000	3.82 ± 2.01	3.09 ± 1.62	−6.691	0.000
ABSI	8.22 ± 0.53	8.17 ± 0.54	−1.331	0.183	8.28 ± 0.60	8.20 ± 0.58	−2.404	0.016
BRI	3.97 ± 0.93	3.31 ± 0.83	−10.726	0.000	4.46 ± 1.28	3.85 ± 1.14	−8.677	0.000
LAP	27.54 ± 16.43	16.51 ± 11.92	−10.289	0.000	30.53 ± 15.00	22.18 ± 12.15	−10.307	0.000
CI	1.28 ± 0.08	1.25 ± 0.08	−5.376	0.000	1.29 ± 0.09	1.26 ± 0.09	−5.537	0.000
CVAI	103.07 ± 33.98	71.63 ± 33.02	−14.083	0.000	95.43 ± 31.37	73.93 ± 30.11	−12.512	0.000
TyG index	8.56 ± 0.45	8.35 ± 0.46	−6.702	0.000	8.46 ± 0.43	8.33 ± 0.42	−5.609	0.000
TyG-BMI	203.92 ± 27.27	180.57 ± 26.96	−12.842	0.000	201.90 ± 33.12	185.10 ± 30.85	−9.460	0.000
TyG-WC	744.31 ± 78.26	673.52 ± 74.19	−14.069	0.000	714.95 ± 84.11	663.65 ± 78.44	−11.369	0.000
TyG-WHtR	4.53 ± 0.46	4.13 ± 0.45	−13.028	0.000	4.67 ± 0.56	4.35 ± 0.51	−10.940	0.000

The data are presented either as the mean ± SD or *n* (%).

WC, waist circumference; BMI, body mass index; WHtR, waist-to-height ratio; VAI, visceral adiposity index; ABSI, a body shape index; BRI, body roundness index; LAP, lipid accumulation product; CVAI, cardio-ankle vascular index; CI, conicity index; TyG, triglyceride and glucose index; TyG-BMI, TyG related to BMI; TyG-WC, TyG related to WC; TyG-WHtR, TyG related to WHtR.

## Results

Table 1 shows the baseline characteristics of participants according to gender differences. The number of participants was 3,640; 54.07% were men and 45.93% were women. Notably, 10.27% were single; 94.59% were living in rural areas; 8.30% were former smokers and 36.13% were current smokers; 8.27% were drinking less than once a month and 29.12% were drinking more than once a month; 48.13% were taking activities; 50.05% had 1–2 chronic diseases and 14.56% had 3–14 chronic diseases. Meanwhile, the current residence, taking activities, doing regular exercises, and chronic disease counts were not statistically significant between the male and female subgroups (*P* > 0.05).

Table 2 shows the baseline characteristics of the study participants with and without MetS by sex. A total of 3,640 participants were classified into two groups according to sex. A total of 1,968 men, robust with MetS (12.86%) and without MetS (87.14%) at baseline, were included in the national cohort analysis. A total of 1,672 women, robust with MetS (25.00%) and without MetS (75.00%) at baseline, were included in the national cohort analysis. There was no significant difference in ABSI between subgroups of patients with and without MetS, whether in men or women (*P* > 0.05). At the same time, the remaining 12 indicators differed between subgroups of patients with and without MetS (*P* < 0.05).

**Figure 3:**
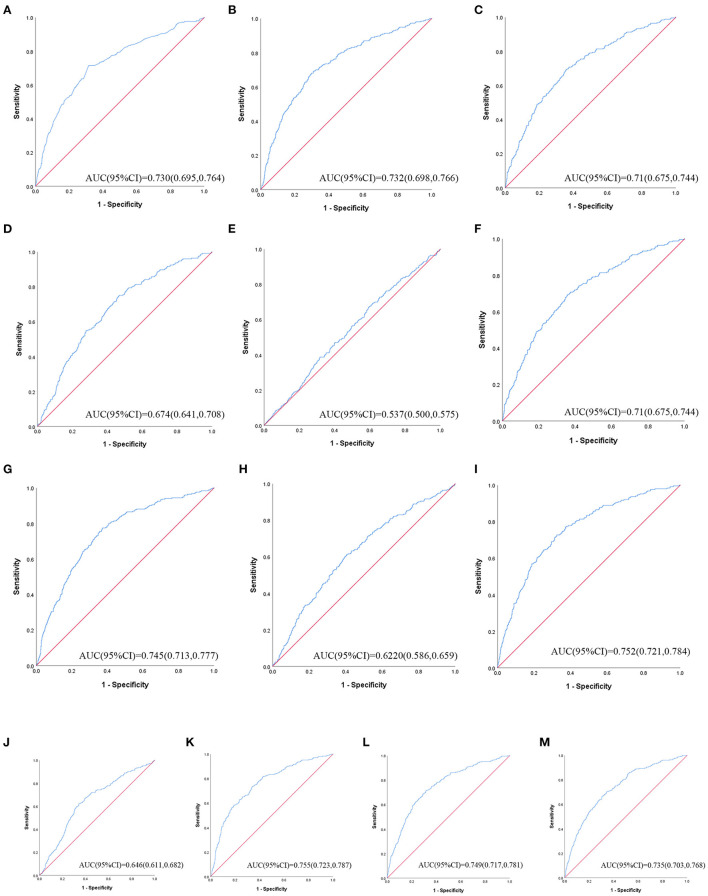
The ROC curves of each indicator in the prediction of MetS risk in male. **(A)** = WC, **(B)** = BMI, **(C)** = WHtR, **(D)** = VAI, **(E)** = ABSI, **(F)** = BRI, **(G)** = LAP, **(H)** = CI, **(I)** = CVAI, **(J)** = TyG-index, **(K)** = TyG-BMI, **(L)** = TyG-WC, **(M)** = TyG-WHtR.

**Figure 4:**
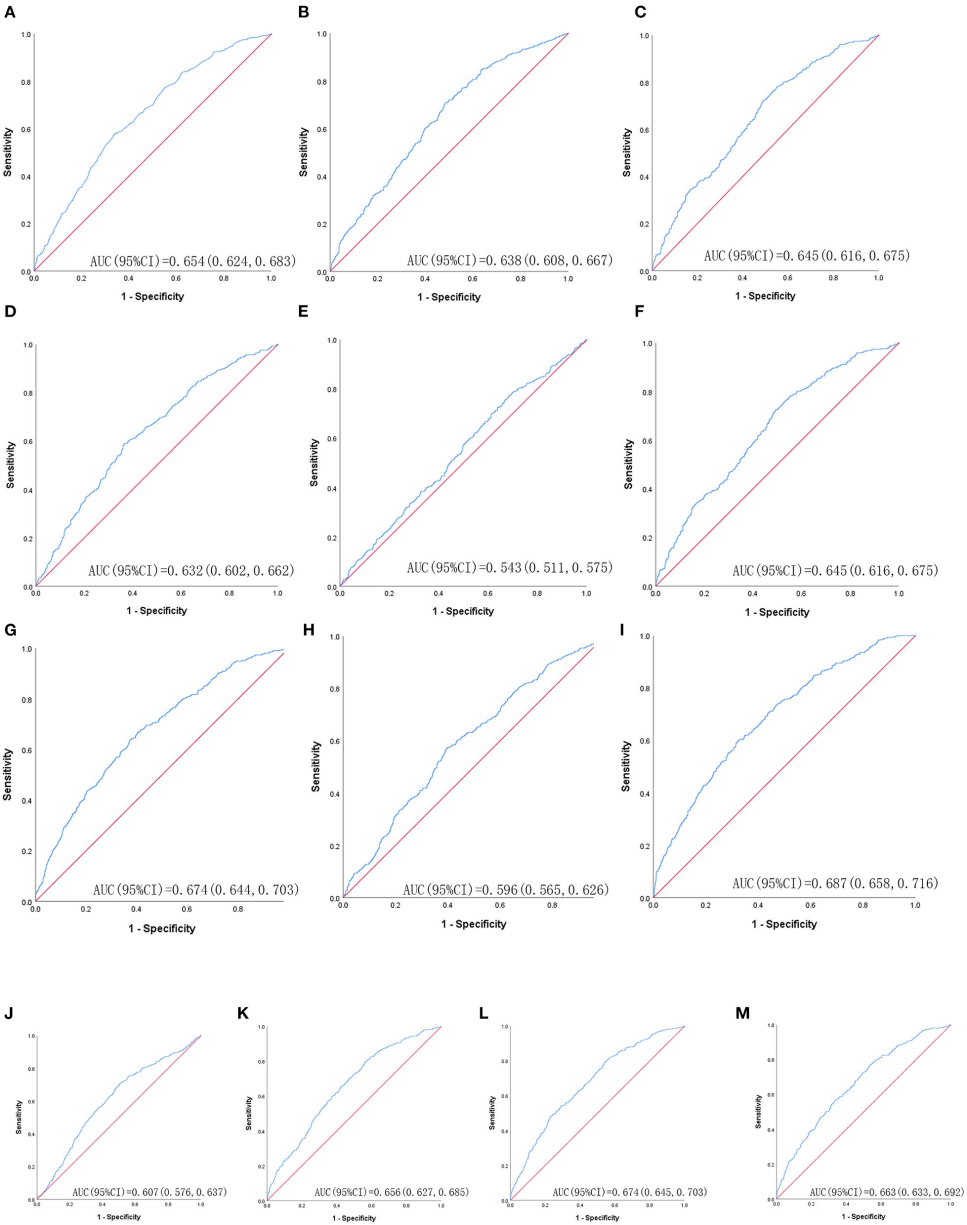
The ROC curves of each indicator in the prediction of MetS risk in female. **(A)** = WC, **(B)** = BMI, **(C)** = WHtR, **(D)** = VAI, **(E)** = ABSI, **(F)** = BRI, **(G)** = LAP, **(H)** = CI, **(I)** = CVAI, **(J)** = TyG-index, **(K)** = TyG-BMI, **(L)** = TyG-WC, **(M)** = TyG-WHtR.

Table 3 shows ROC analysis and AUC results of obesity- and lipid-related indices. We observed the predictive value of obesity- and lipid-related indicators for MetS by sex using the ROC. The ROC curves of each indicator in the prediction of MetS risk in men and women are shown in Figures 3, 4, respectively. In men, the largest AUC was observed for the TyG-BMI index (AUC = 0.755, Std. Error = 0.016, 95% CI = 0.723–0.787, and optimal cutoff value = 187.919). The prediabetes predictive values were similar to the CVAI (AUC = 0.752, Std. Error = 0.016, 95% CI = 0.721–0.784, and optimal cutoff value = 85.284), TyG-WC (AUC = 0.749, Std. Error = 0.016, 95% CI = 0.717–0.781, and optimal cutoff value = 705.405), and LAP (AUC = 0.745, Std. Error = 0.016, 95% CI = 0.713–0.777, and optimal cutoff value = 16.896) indices. In contrast, the AUC of ABSI did not reach statistical significance (*P* = 0.055). In women, the largest AUC was observed for the CVAI index (AUC = 0.687, Std. Error = 0.015, 95% CI = 0.658–0.716, and optimal cutoff value = 86.785). The prediabetes predictive values were similar to the LAP (AUC = 0.674, Std. Error = 0.015, 95% CI = 0.644–0.703, and optimal cutoff value = 23.723), TyG-WC (AUC = 0.674, Std. Error = 0.015, 95% CI = 0.645–0.703, and optimal cutoff value = 644.630), and TyG-WHtR (AUC = 0.663, Std. Error = 0.015, 95% CI = 0.633–0.692, and optimal cutoff value = 4.265) indices. As in men, the AUC of ABSI in women was not statistically significant (*P* = 0.009).

**Table 3:** Cutoff value between area under curve, sensitivity, and specificity for obesity- and lipid-related indices to detect metabolic syndrome (CHARLS2011–2015) by sex.

***N* = 3,088**	**WC**	**BMI**	**WHtR**	**VAI**	**ABSI**	**BRI**	**LAP**	**CI**	**CVAI**	**TyG index**	**TyG-BMI**	**TyG-WC**	**TyG-WHtR**
**Male (*****N*** = **1,968)**													
Area under curve	0.730	0.732	0.710	0.674	0.537	0.710	0.745	0.622	0.752	0.646	0.755	0.749	0.735
Std. error	0.018	0.017	0.017	0.017	0.019	0.017	0.016	0.019	0.016	0.018	0.016	0.016	0.017
95% CI	0.695, 0.764	0.698, 0.766	0.675, 0.744	0.641, 0.708	0.500, 0.575	0.675, 0.744	0.713, 0.777	0.586, 0.659	0.721, 0.784	0.611, 0.682	0.723, 0.787	0.717, 0.781	0.703, 0.768
*P*-value	0.000	0.000	0.000	0.000	0.055	0.000	0.000	0.000	0.000	0.000	0.000	0.000	0.000
Optimal cutoffs	83.950	22.569	0.509	2.030	8.054	3.523	16.896	1.264	85.284	8.493	187.919	705.405	4.342
J-Youden	0.400	0.384	0.336	0.283	0.079	0.335	0.402	0.208	0.402	0.282	0.395	0.399	0.399
Sensitivity (%)	0.715	0.692	0.692	0.751	0.672	0.692	0.775	0.613	0.727	0.625	0.735	0.715	0.668
Specificity (%)	0.685	0, 692	0.644	0.532	0.407	0.644	0.627	0.595	0.675	0.657	0.660	0.683	0.694
(+) Likelihood ratio	2.270	2.247	1.944	1.605	1.133	1.942	2.079	1.514	2.239	1.821	2.162	2.260	2.183
(–) Likelihood ratio	0.416	0.445	0.478	0.468	0.806	0.479	0.359	0.650	0.404	0.571	0.402	0.416	0.478
**Female (*****N*** = **1,672)**													
Area under curve	0.654	0.638	0.645	0.632	0.543	0.645	0.674	0.596	0.687	0.607	0.656	0.674	0.663
Std. error	0.015	0.015	0.015	0.015	0.016	0.015	0.015	0.016	0.015	0.016	0.015	0.015	0.015
95% CI	0.624, 0.683	0.608, 0.667	0.616, 0.675	0.602, 0.662	0.511, 0.575	0.616, 0.675	0.644, 0.703	0.565, 0.626	0.658, 0.716	0.576, 0.637	0.627, 0.685	0.645, 0.703	0.633, 0.692
*P*-value	0.000	0.000	0.000	0.000	0.009	0.000	0.000	0.000	0.000	0.000	0.000	0.000	0.000
Optimal cutoffs	82.700	21.923	0.517	3.244	7.918	3.680	23.723	1.275	86.785	8.297	187.164	644.630	4.265
J-Youden	0.240	0.224	0.231	0.225	0.089	0.231	0.264	0.175	0.276	0.203	0.233	0.252	0.235
Sensitivity (%)	0.574	0.708	0.725	0.589	0.775	0.725	0.644	0.574	0.603	0.711	0.656	0.821	0.773
Specificity (%)	0.666	0.516	0.506	0.636	0.313	0.506	0.620	0.601	0.673	0.492	0.577	0.431	0.462
(+) Likelihood ratio	1.719	1.463	1.468	1.618	1.129	1.468	1.695	1.439	1.844	1.400	1.551	1.443	1.437
(–) Likelihood ratio	0.640	0.566	0.543	0.646	0.718	0.543	0.574	0.709	0.590	0.587	0.596	0.416	0.491

WC, waist circumference; BMI, body mass index; WHtR, waist to height ratio; VAI, visceral adiposity index; ABSI, a body shape index; BRI, body roundness index; LAP, lipid accumulation product; CVAI, cardio-ankle vascular index; CI, conicity index; TyG, triglyceride and glucose index; TyG-BMI, TyG related to BMI; TyG-WC, TyG related to WC; TyG-WHtR, TyG related to WHtR.

**Figure 5:**
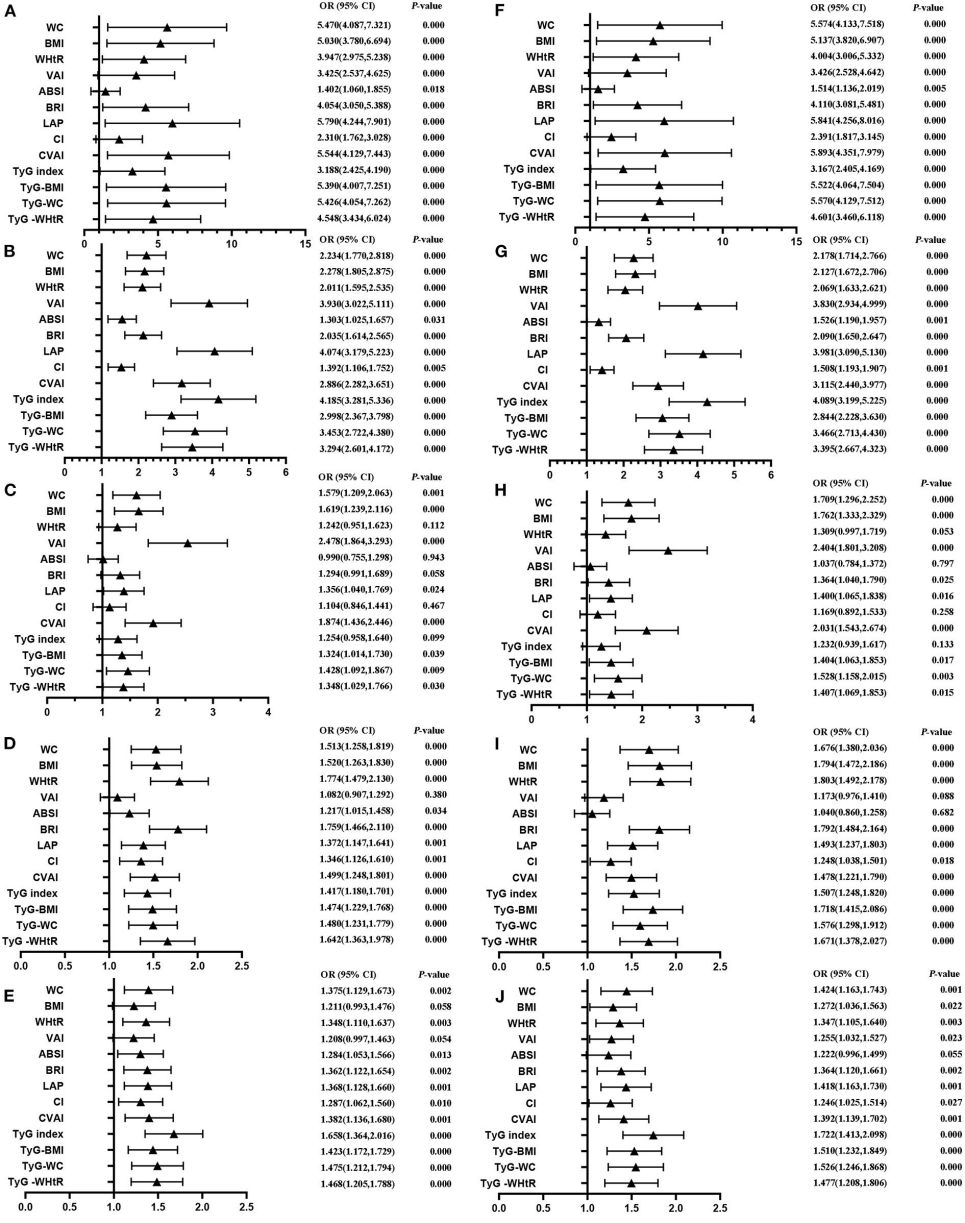
Forest diagram of OR before and after adjustment of confounding factors for male. **(A)** Mets unadjust, **(B)** Elevated triglycerides unadjusted, **(C)** Reduced HDL-C unadjusted, **(D)** Elevated blood pressure unadjusted, **(E)** Elevated fasting glucose unadjusted, **(F)** Mets adjusted, **(G)** Elevated triglycerides adjusted, **(H)** Reduced HDL-C adjusted, **(I)** Elevated blood pressure adjusted, **(J)** Elevated fasting glucose adjusted. Adjusted OR: Adjusted for age, educational levels, marital status, live place, current smoking, alcohol drinking, activities, exercises, chronic diseases.

**Figure 6:**
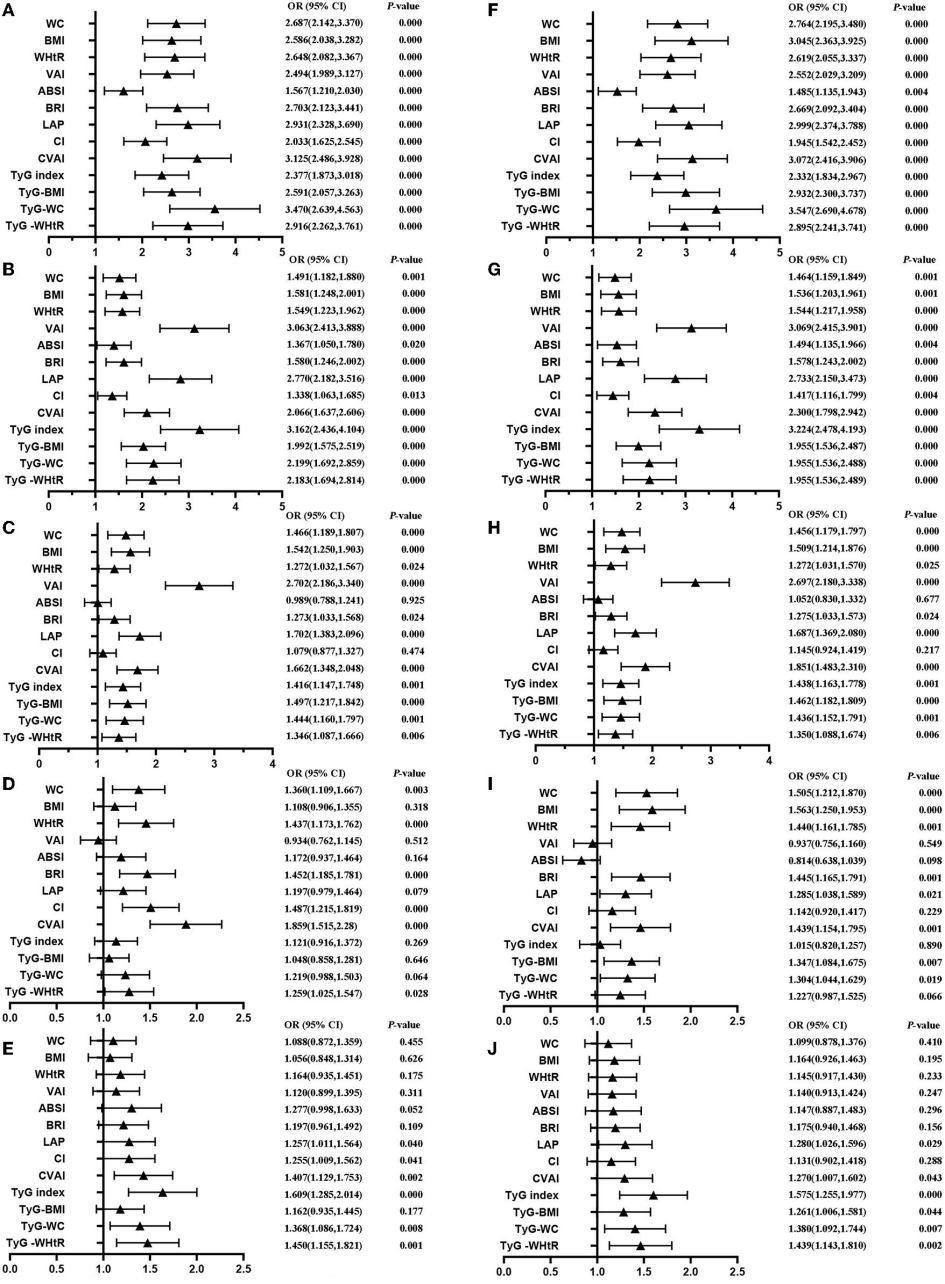
Forest diagram of OR before and after adjustment of confounding factors for female. **(A)** Mets unadjust, **(B)** Elevated triglycerides unadjusted, **(C)** Reduced HDL-C unadjusted, **(D)** Elevated blood pressure unadjusted, **(E)** Elevated fasting glucose unadjusted, **(F)** Mets adjusted, **(G)** Elevated triglycerides adjusted, **(H)** Reduced HDL-C adjusted, **(I)** Elevated blood pressure adjusted, **(J)** Elevated fasting glucose adjusted. Adjusted OR: Adjusted for age, educational levels, marital status, live place, current smoking, alcohol drinking, activities, exercises, chronic diseases.

Table 4 shows that MetS and its components increase gradually with the increase of obesity- and lipid-related indices in both sexes. For instance, in men, a unit increase in WC was associated with a 5.574-fold increased odds of metabolic syndrome (aOR: 5.574; 95% CI: 4.133–7.518), and a unit increase in BMI was associated with a 5.137-fold increase in odds of metabolic syndrome (aOR: 5.137; 95% CI: 3.820–6.907). In women, a unit increase in WC was associated with a 2.764-fold increase in odds of metabolic syndrome (aOR: 2.764; 95% CI: 2.195–3.480), and a unit increase in BRI was associated with a 3.045-fold increased odds of metabolic syndrome (aOR: 3.045; 95% CI: 2.363-3.925). The OR and 95% CI of Table 4 are used to draw Figures 5, 6. It is interesting that OR values were generally higher in men than in women for risk factors of MetS. Meanwhile, the OR value of ABSI was much lower than that of the other 13 indices.

**Table 4:** Associations of obesity- and lipid-related indices with MetS (CHARLS2011–2015) and its components.

**MetS and its components**	**WC**	**BMI**	**WHtR**	**VAI**	**ABSI**	**BRI**	**LAP**	**CI**	**CVAI**	**TyG index**	**TyG-BMI**	**TyG-WC**	**TyG-WHtR**
**Male**													
**MetS**													
Unadjusted OR (95% CI)	5.470 (4.087, 7.321)^**^	5.030 (3.780, 6.694)^**^	3.947 (2.975, 5.238)^**^	3.425 (2.537, 4.625)^**^	1.402 (1.060, 1.855)^*^	4.054 (3.050, 5.388)^**^	5.790 (4.244, 7.901)^**^	2.310 (1.762, 3.028)^**^	5.544 (4.129, 7.443)^**^	3.188 (2.425, 4.190)^**^	5.390 (4.007, 7.251)^**^	5.426 (4.054, 7.262)^**^	4.548 (3.434, 6.024)^**^
*P*-value	0.000	0.000	0.000	0.000	0.018	0.000	0.000	0.000	0.000	0.000	0.000	0.000	0.000
Adjusted OR (95% CI)	5.574 (4.133, 7.518)^**^	5.137 (3.820, 6.907)^**^	4.004 (3.006, 5.332)^**^	3.426 (2.528, 4.642)^**^	1.514 (1.136, 2.019)^*^	4.110 (3.081, 5.481)^**^	5.841 (4.256, 8.016)^**^	2.391 (1.817, 3.145)^**^	5.893 (4.351, 7.979)^**^	3.167 (2.405, 4.169)^**^	5.522 (4.064, 7.504)^**^	5.570 (4.129, 7.512)^**^	4.601 (3.460, 6.118)^**^
*P*-value	0.000	0.000	0.000	0.000	0.005	0.000	0.000	0.000	0.000	0.000	0.000	0.000	0.000
**Elevated triglycerides**													
Unadjusted OR (95% CI)	2.234 (1.770, 2.818)^**^	2.278 (1.805, 2.875)^**^	2.011 (1.595, 2.535)^**^	3.930 (3.022, 5.111)^**^	1.303 (1.025, 1.657)^*^	2.035 (1.614, 2.565)^**^	4.074 (3.179, 5.223)^**^	1.392 (1.106, 1.752)^*^	2.886 (2.282, 3.651)^**^	4.185 (3.281, 5.336)^**^	2.998 (2.367, 3.798)^**^	3.453 (2.722, 4.380)^**^	3.294 (2.601, 4.172)^**^
*P*-value	0.000	0.000	0.000	0.000	0.031	0.000	0.000	0.005	0.000	0.000	0.000	0.000	0.000
Adjusted OR (95% CI)	2.178 (1.714, 2.766)^**^	2.127 (1.672, 2.706)^**^	2.069 (1.633, 2.621)^**^	3.830 (2.934, 4.999)^**^	1.526 (1.190, 1.957)^**^	2.090 (1.650, 2.647)^**^	3.981 (3.090, 5.130)^**^	1.508 (1.193, 1.907)^**^	3.115 (2.440, 3.977)^**^	4.089 (3.199, 5.225)^**^	2.844 (2.228, 3.630)^**^	3.466 (2.713, 4.430)^**^	3.395 (2.667, 4.323)^**^
*P*-value	0.000	0.000	0.000	0.000	0.001	0.000	0.000	0.001	0.000	0.000	0.000	0.000	0.000
**Reduced HDL-C**													
Unadjusted OR (95% CI)	1.579 (1.209, 2.063)^**^	1.619 (1.239, 2.116)^**^	1.242 (0.951, 1.623)	2.478 (1.864, 3.293)^**^	0.990 (0.755, 1.298)	1.294 (0.991, 1.689)	1.356 (1.040, 1.769)^*^	1.104 (0.846, 1.441)	1.874 (1.436, 2.446)^**^	1.254 (0.958, 1.640)	1.324 (1.014, 1.730)^*^	1.428 (1.092, 1.867)^*^	1.348 (1.029, 1.766)^*^
*P*-value	0.001	0.000	0.112	0.000	0.943	0.058	0.024	0.467	0.000	0.099	0.039	0.009	0.030
Adjusted OR (95% CI)	1.709 (1.296, 2.252)^**^	1.762 (1.333, 2.329)^**^	1.309 (0.997, 1.719)	2.404 (1.801, 3.208)^**^	1.037 (0.784, 1.372)	1.364 (1.040, 1.790)^*^	1.400 (1.065, 1.838)^*^	1.169 (0.892, 1.533)	2.031 (1.543, 2.674)^**^	1.232 (0.939, 1.617)	1.404 (1.063, 1.853)^*^	1.528 (1.158, 2.015)^*^	1.407 (1.069, 1.853)^*^
*P*-value	0.000	0.000	0.053	0.000	0.797	0.025	0.016	0.258	0.000	0.133	0.017	0.003	0.015
**Elevated blood pressure**													
Unadjusted OR (95% CI)	1.513 (1.258, 1.819)^**^	1.520 (1.263, 1.830)^**^	1.774 (1.479, 2.130)^**^	1.082 (0.907, 1.292)	1.217 (1.015, 1.458)^*^	1.759 (1.466, 2.110)^**^	1.372 (1.147, 1.641)^**^	1.346 (1.126, 1.610)^*^	1.499 (1.248, 1.801)^**^	1.417 (1.180, 1.701)^**^	1.474 (1.229, 1.768)^**^	1.480 (1.231, 1.779)^**^	1.642 (1.363, 1.978)^**^
*P*-value	0.000	0.000	0.000	0.380	0.034	0.000	0.001	0.001	0.000	0.000	0.000	0.000	0.000
Adjusted OR (95% CI)	1.676 (1.380, 2.036)^**^	1.794 (1.472, 2.186)^**^	1.803 (1.492, 2.178)^**^	1.173 (0.976, 1.410)	1.040 (0.860, 1.258)	1.792 (1.484, 2.164)^**^	1.493 (1.237, 1.803)^**^	1.248 (1.038, 1.501)^*^	1.478 (1.221, 1.790)^**^	1.507 (1.248, 1.820)^**^	1.718 (1.415, 2.086)^**^	1.576 (1.298, 1.912)^**^	1.671 (1.378, 2.027)^**^
*P*-value	0.000	0.000	0.000	0.088	0.682	0.000	0.000	0.018	0.000	0.000	0.000	0.000	0.000
**Elevated fasting glucose**													
Unadjusted OR (95% CI)	1.375 (1.129, 1.673)^*^	1.211 (0.993, 1.476)	1.348 (1.110, 1.637)^*^	1.208 (0.997, 1.463)	1.284 (1.053, 1.566)^*^	1.362 (1.122, 1.654)^*^	1.368 (1.128, 1.660)^*^	1.287 (1.062, 1.560)^*^	1.382 (1.136, 1.680)^*^	1.658 (1.364, 2.016)^**^	1.423 (1.172, 1.729)^**^	1.475 (1.212, 1.794)^**^	1.468 (1.205, 1.788)^**^
*P*-value	0.002	0.058	0.003	0.054	0.013	0.002	0.001	0.010	0.001	0.000	0.000	0.000	0.000
*P*-value	0.001	0.022	0.003	0.023	0.055	0.002	0.001	0.027	0.001	0.000	0.000	0.000	0.000
**Female**													
**MetS**													
Unadjusted OR (95% CI)	2.687 (2.142, 3.370)^**^	2.586 (2.038, 3.282)^**^	2.648 (2.082, 3.367)^**^	2.494 (1.989, 3.127)^**^	1.567 (1.210, 2.030)^**^	2.703 (2.123, 3.441)^**^	2.931 (2.328, 3.690)^**^	2.033 (1.625, 2.545)^**^	3.125 (2.486, 3.928)^**^	2.377 (1.873, 3.018)^**^	2.591 (2.057, 3.263)^**^	3.470 (2.639, 4.563)^**^	2.916 (2.262, 3.761)^**^
*P*-value	0.000	0.000	0.000	0.000	0.001	0.000	0.000	0.000	0.000	0.000	0.000	0.000	0.000
Adjusted OR (95% CI)	2.764 (2.195, 3.480)^**^	3.045 (2.363, 3.925)^**^	2.619 (2.055, 3.337)^**^	2.552 (2.029, 3.209)^**^	1.485 (1.135, 1.943)^*^	2.669 (2.092, 3.404)^**^	2.999 (2.374, 3.788)^**^	1.945 (1.542, 2.452)^**^	3.072 (2.416, 3.906)^**^	2.332 (1.834, 2.967)^**^	2.932 (2.300, 3.737)^**^	3.547 (2.690, 4.678)^**^	2.895 (2.241, 3.741)^**^
*P*-value	0.000	0.000	0.000	0.000	0.004	0.000	0.000	0.000	0.000	0.000	0.000	0.000	0.000
**Elevated triglycerides**													
Unadjusted OR (95% CI)	1.491 (1.182, 1.88)^**^	1.581 (1.248, 2.001)^**^	1.549 (1.223, 1.962)^**^	3.063 (2.413, 3.888)^**^	1.367 (1.050, 1.780)^*^	1.580 (1.246, 2.002)^**^	2.770 (2.182, 3.516)^**^	1.338 (1.063, 1.685)^*^	2.066 (1.637, 2.606)^**^	3.162 (2.436, 4.104)^**^	1.992 (1.575, 2.519)^**^	2.199 (1.692, 2.859)^**^	2.183 (1.694, 2.814)^**^
*P*-value	0.001	0.000	0.000	0.000	0.020	0.000	0.000	0.013	0.000	0.000	0.000	0.000	0.000
Adjusted OR (95% CI)	1.464 (1.159, 1.849)^*^	1.536 (1.203, 1.961)^**^	1.544 (1.217, 1.958)^**^	3.069 (2.415, 3.901)^**^	1.494 (1.135, 1.966)^*^	1.578 (1.243, 2.002)^**^	2.733 (2.15, 3.473)^**^	1.417 (1.116, 1.799)^*^	2.300 (1.798, 2.942)^**^	3.224 (2.478, 4.193)^**^	1.955 (1.536, 2.487)^**^	2.174 (1.670, 2.829)^**^	2.183 (1.691, 2.819)^**^
*P*-value	0.001	0.001	0.000	0.000	0.004	0.000	0.000	0.004	0.000	0.000	0.000	0.000	0.000
**Reduced HDL-C**													
Unadjusted OR (95% CI)	1.466 (1.189, 1.807)^**^	1.542 (1.25, 1.903)^**^	1.272 (1.032, 1.567)^*^	2.702 (2.186, 3.34)^**^	0.989 (0.788, 1.241)	1.273 (1.033, 1.568)^*^	1.702 (1.383, 2.096)^**^	1.079 (0.877, 1.327)	1.662 (1.348, 2.048)^**^	1.416 (1.147, 1.748)^*^	1.497 (1.217, 1.842)^**^	1.444 (1.16, 1.797)^*^	1.346 (1.087, 1.666)^*^
*P*-value	0.000	0.000	0.024	0.000	0.925	0.024	0.000	0.474	0.000	0.001	0.000	0.001	0.006
Adjusted OR (95% CI)	1.456 (1.179, 1.797)^**^	1.509 (1.214, 1.876)^**^	1.272 (1.031, 1.570)^*^	2.697 (2.18, 3.338)^**^	1.052 (0.83, 1.332)	1.275 (1.033, 1.573)^*^	1.687 (1.369, 2.080)^**^	1.145 (0.924, 1.419)	1.851 (1.483, 2.310)^**^	1.438 (1.163, 1.778)^**^	1.462 (1.182, 1.809)^**^	1.436 (1.1520, 1.791)^*^	1.350 (1.088, 1.674)^*^
*P*-value	0.000	0.000	0.025	0.000	0.677	0.024	0.000	0.217	0.000	0.001	0.000	0.001	0.006
**Elevated blood pressure**													
Unadjusted OR (95% CI)	1.360 (1.109, 1.667)^*^	1.108 (0.906, 1.355)	1.437 (1.173, 1.762)^**^	0.934 (0.762, 1.145)	1.172 (0.937, 1.464)	1.452 (1.185, 1.781)^**^	1.197 (0.979, 1.464)	1.487 (1.215, 1.819)^**^	1.859 (1.515, 2.28)^**^	1.121 (0.916, 1.372)	1.048 (0.858, 1.281)	1.219 (0.988, 1.503)	1.259 (1.025, 1.547)^*^
*P*-value	0.003	0.318	0.000	0.512	0.164	0.000	0.079	0.000	0.000	0.269	0.646	0.064	0.028
Adjusted OR (95% CI)	1.505 (1.212, 1.870)^**^	1.563 (1.250, 1.953)^**^	1.44 (1.161, 1.785)^**^	0.937 (0.756, 1.160)	0.814 (0.638, 1.039)	1.445 (1.165, 1.791)^**^	1.285 (1.038, 1.589)^*^	1.142 (0.920, 1.417)	1.439 (1.154, 1.795)^*^	1.015 (0.82, 1.257)	1.347 (1.084, 1.675)^*^	1.304 (1.044, 1.629)^*^	1.227 (0.987, 1.525)
*P*-value	0.000	0.000	0.001	0.549	0.098	0.001	0.021	0.229	0.001	0.890	0.007	0.019	0.066
**Elevated fasting glucose**													
Unadjusted OR (95% CI)	1.088 (0.872, 1.359)	1.056 (0.848, 1.314)	1.164 (0.935, 1.451)	1.12 (0.899, 1.395)	1.277 (0.998, 1.633)	1.197 (0.961, 1.492)	1.257 (1.011, 1.564)^*^	1.255 (1.009, 1.562)^*^	1.407 (1.129, 1.753)^*^	1.609 (1.285, 2.014)^**^	1.162 (0.935, 1.445)	1.368 (1.086, 1.724)^*^	1.45 (1.155, 1.821)^*^
*P*-value	0.455	0.626	0.175	0.311	0.052	0.109	0.040	0.041	0.002	0.000	0.177	0.008	0.001
Adjusted OR (95% CI)	1.099 (0.878, 1.376)	1.164 (0.926, 1.463)	1.145 (0.917, 1.43)	1.14 (0.913, 1.424)	1.147 (0.887, 1.483)	1.175 (0.94, 1.468)	1.28 (1.026, 1.596)^*^	1.131 (0.902, 1.418)	1.27 (1.007, 1.602)^*^	1.575 (1.255, 1.977)^**^	1.261 (1.006, 1.581)^*^	1.38 (1.092, 1.744)^*^	1.439 (1.143, 1.81)^*^
*P*-value	0.410	0.195	0.233	0.247	0.296	0.156	0.029	0.288	0.043	0.000	0.044	0.007	0.002

The obesity- and lipid-related indices (WC, BMI, WHtR, VAI, ABSI, BRI, LAP, CVAI, CI, TyG, TyG-BMI, TyG-WC, and TyG-WHtR) used data from participants in 2011. The MetS components (elevated triglycerides, reduced HDL-C, elevated blood pressure, elevated fasting glucose) used data from participants in 2015.

Adjusted OR: adjusted for age, educational levels, marital status, live place, current smoking, alcohol drinking, activities, exercises, chronic diseases.

* *P* < 0.05,

** *P* < 0.001.

WC, waist circumference; BMI, body mass index; WHtR, waist-to-height ratio; VAI, visceral adiposity index; ABSI, a body shape index; BRI, body roundness index; LAP, lipid accumulation product; CVAI, cardio-ankle vascular index; CI, conicity index; TyG, triglyceride and glucose index; TyG-BMI, TyG related to BMI; TyG-WC, TyG related to WC; TyG-WHtR, TyG related to WHtR.

## Discussion

According to the diagnostic criteria of MetS issued in 2006, this study evaluated the predictive power of 13 obesity- and lipid-related parameters to identify the risk of MetS in middle-aged and elderly Chinese adults. This article is an original study. To the best of our knowledge, no previous study has compared the ability of these 13 obesity- and lipid-related factors to predict MetS in middle-aged and elderly Chinese populations. There are 3,640 participants in this study. Among 3,640 individuals, the prevalence of MetS was 18.43%. Among 1,968 male participants, 253 (12.86%) were diagnosed with MetS. Among 1,672 female participants, 418 (25.00%) were diagnosed with MetS. According to the meta-analysis report (1), the prevalence of MetS in China was 24.5% in 2016, among which the prevalence of MetS increases with age (15–39 years: 13.9%; 40–59 years: 26.4%; 60 years: 32.4%). Ranasinghe et al.'s (16) research report says that the prevalence of MetS in China is increasing yearly. Based on these studies, further studies are needed to establish a single index for predicting the occurrence of MetS in the middle-aged and elderly population in China.

This national cohort study found that the correlation between ABSI and MetS was far lower than the other 12 obesity- and lipid-related indices. Through ROC analysis of obesity- and blood lipid-related indicators, the results for the ABSI AUC did not reach statistical significance (*P* = 0.055) in men, similar to the results (*P*= 0.009) obtained in women. At the same time, some studies (56–58) also show that BRI is better than ABSI in predicting MetS. According to Ji et al. (59), this result can be explained by the following viewpoints: ABSI is highly clustered around the mean value with relatively slight variance, which makes ABSI perform poorly in predicting chronic diseases.

Among the other 12 indices, BMI, WHtR, WC, LAP, and CVAI have received special attention. Ashwell et al. (60) reported that WHtR outperformed WC and BMI in detecting cardiac metabolic risk factors in both men and women. Similarly, in a systematic literature review in 2016, Corrêa et al. (61) also shows that WHtR has better performance than WC and BMI in evaluating MetS. Alves et al.'s (62) study shows that LAP, WC, and WHtR indicators identify MetS in older women and can be used to assess and monitor MetS individually or collectively.

Tyg-related factors such as the TyG index, TYG-BMI, TYG-WC, and TYG-WHTR values may provide a broader basis for assessing the association between MetS and obesity- and lipid-related indicators. Thus, TyG-related factors have also recently been suggested as a method for estimating metabolic disorders. Ahn et al.'s (63) study shows that the TyG index provided a good standard for identifying people with pre-diabetes/diabetes. In the meantime, most of the studies (64–66) also showed a significant correlation between TyG-related factors and pre-diabetes/diabetes. As mentioned earlier, elevated FPG levels were one of the diagnostic criteria for MetS, and pre-diabetes/diabetes leads to elevated FPG levels. Therefore, there must be an association between TyG-related factors and MetS. Ferreira et al. (67) identified the TyG-related factors as an important tool for predicting MetS.

Indeed, in this study, the TyG-related factors were more efficient compared to these other markers. According to the results of this study, obesity- and lipid-related indices are different in predicting MetS because of gender differences. In general, an AUC closer to 1 indicates better predictive power, while an AUC of 0.55 or less indicates no better predictive power than chance. Among men, TyG-BMI performed best, followed by CVAI, TyG-WC, and LAP. TyG-BMI is a surrogate marker used to identify insulin resistance (68). In the ROC analysis of obesity- and blood lipid-related indicators, the largest AUC was observed for the TyG-BMI index (AUC = 0.755, Std. Error = 0.016, 95% CI = 0.723–0.787, and optimal cutoff value = 187.919). Raimi et al. (69) suggested that the product of TyG-related factors and the anthropometric index could improve the identification and prediction of MetS. This view is consistent with this article.

In contrast, according to the report (70), VAI has moderate to high accuracy in screening MetS diagnostic tests. VAI was a reliable index to evaluate the visceral fat function, as proposed by Amato et al. (46). A multi-center cross-sectional study (58) was conducted in Guizhou Province in southwest China, and it was found that LAP and VAI, the visceral obesity markers, were the most effective predictors of MetS, while ABSI and CI were the weakest indicators for screening MetS. Interestingly, many studies (71, 72) have also demonstrated that VAI performs well in predicting MetS.

Considerable evidence suggests that VAI plays a significant role in predicting MetS (73, 74). However, there were significant differences in body fat distribution between ethnic groups. According to the distribution characteristics of Asian body fat, Xia et al. developed CVAI to evaluate the visceral fat area of the Chinese people (51). CVAI is better than VAI in predicting the MetS in middle-aged and older adults in China. Among women, CVAI performed best, followed by LAP, TyG-WC, and TyG-WHtR. In the ROC analysis of obesity- and blood lipid-related indicators, the largest AUC was observed for the CVAI index (AUC = 0.687, Std. Error = 0.015, 95% CI = 0.658–0.716, and optimal cutoff value = 86.785).

Similarly, LAP performed well in predicting metabolic synthesis. LAP was used as an indicator to predict insulin resistance (75, 76), so there is also a significant association between LAP and MetS. In this study, for men, the AUC of LAP (AUC = 0.745, Std. Error = 0.016, 95% CI = 0.713–0.777, and optimal cutoff value = 16.896) was the largest of the other factors in the ROC analysis of obesity- and lipid-related measures, except for the TyG-related factors and CVAI. For women, the AUC of LAP (AUC = 0.674, Std. Error = 0.015, 95% CI = 0.644–0.703, and optimal cutoff value = 23.723) was second only to that of CVAI among the other factors. The rest of the studies (77–79) also showed that LAP works well in predicting MetS.

In this study, 13 obesity- and lipid-related indices were converted into two-category variables according to the values in Table 3. Table 4 is based on the transformed variables. In general, a higher OR indicates a greater risk factor. In Table 4, the OR value of ABSI is much lower than that of the remaining 12 indices in both sexes, which is consistent with the conclusion drawn in Table 3. ABSI is not suitable as a predictor of MetS. Stefanescu A showed in the study that ABSI is much inferior to other indicators for predicting MetS in Peruvian adults (80). Khan et al.'s study showed that WHtR and VAI outperformed ABSI in predicting MetS (22). At the same time, many studies (22, 81, 82) are consistent with the viewpoint of this study. It is interesting that the OR value of men is greater than that of women before and after adjustment. This suggests that an increase in obesity factors may make men more susceptible to MetS. There are many reasons for this. Men are more vulnerable to tobacco damage than women. Many studies (83, 84) have shown that tobacco is more likely to cause sugar and lipid metabolism disorders in the elderly. This may be associated with cigarette smoke impairing the reverse transport of cholesterol (85). At the same time, men are more prone to alcohol abuse than women in daily life. Excessive drinking can significantly increase the risk of MetS (86).

## Strengths and limitations of the study

This study has several advantages. This study was based on a nationwide cohort study of middle-aged and older community residents, with participants aged 45 years or older. It compared the effect of different obesity- and lipid-related indices of the MetS and its component symptom. Previous studies have used only a set of single indices to predict the incidence of MetS. It helped us to understand the different obesity- and lipid-related indices on the incidence of MetS. There are several limitations to this study. Many participants were excluded due to missing data, and further studies should gather more complete data.

## Conclusion

Among middle-aged and older adults, all obesity- and lipid-related indices, except ABSI, were able to predict MetS after adjustment for age, sex, educational status, history of smoking, taking activities, doing regular exercises, and chronic diseases. In addition, in men, TyG-BMI is the best indicator to predict MetS, and in women, CVAI is considered the best indicator to predict MetS. LAP had performed well in predicting MetS in both men and women.

## Data availability statement

The original contributions presented in the study are included in the article/supplementary material, further inquiries can be directed to the corresponding author.

## Ethics statement

All data are openly published as microdata at http://charls.pku.edu.cn/ with no direct contact with all participants. Approval for this study was given by the Medical Ethics Committee of Wannan Medical College (approval number 2021–3). The patients/participants provided their written informed consent to participate in this study.

## Author contributions

LZ: conceived the research. JG: wrote the paper and analyzed the data. JG, YLi, HL, LG, JinlL, YLe, XL, LS, LY, TY, CW, DZ, HW, JingL, ML, YH, and LZ: revised the paper. All authors reviewed the manuscript. All authors contributed to the article and approved the submitted version.
